# An Exploratory Study of the Effects of a High School-University Collaborative Program on Learning Motivation and Professional Understanding among Students Aspiring to Enter Medical School

**DOI:** 10.14789/ejmj.JMJ26-0015-OA

**Published:** 2026-04-22

**Authors:** TAKASUKE OGAWA, YUJI NISHIZAKI, MIWA SEKINE, KANAKO OGURA, HAJIME ORITA, YUICHI TOMIKI

**Affiliations:** 1 Division of Medical Education, Juntendo University Faculty of Medicine, Tokyo, Japan; 1 Division of Medical Education, Juntendo University Faculty of Medicine, Tokyo, Japan; 2 Department of Dermatology, Juntendo University Faculty of Medicine, Tokyo, Japan; 2 Department of Dermatology, Juntendo University Faculty of Medicine, Tokyo, Japan; 3 Department of General Medicine, Juntendo University Faculty of Medicine, Tokyo, Japan; 3 Department of General Medicine, Juntendo University Faculty of Medicine, Tokyo, Japan; 4 Department of Cardiovascular Biology and Medicine, Juntendo University Faculty of Medicine, Tokyo, Japan; 4 Department of Cardiovascular Biology and Medicine, Juntendo University Faculty of Medicine, Tokyo, Japan; 5 Department of Microbiology, Juntendo University Faculty of Medicine, Tokyo, Japan; 5 Department of Microbiology, Juntendo University Faculty of Medicine, Tokyo, Japan; 6 Department of Diagnostic Pathology, Juntendo University Nerima Hospital, Tokyo, Japan; 6 Department of Diagnostic Pathology, Juntendo University Nerima Hospital, Tokyo, Japan; 7 Department of Esophageal and Gastroenterological Surgery, Juntendo University Hospital, Tokyo, Japan; 7 Department of Esophageal and Gastroenterological Surgery, Juntendo University Hospital, Tokyo, Japan

**Keywords:** high school-university collaboration, medical education, experiential learning, career development, professional identity formation

## Abstract

**Objective:**

University-high school collaborative programmes for students aspiring to enter medical school may contribute to enhancing learning motivation and career understanding; however, their educational significance has not been sufficiently verified in Japan. This study aimed to clarify the educational significance of a high school-university collaborative programme implemented at Juntendo University.

**Methods:**

A questionnaire survey was conducted among 252 participants in the programme held at Juntendo University’s Hongo Campus between March 2023 and December 2024. The questionnaire assessed enjoyment of the event, understanding of the university, interest in medicine, usefulness for career development, participation in the MEdit class, and satisfaction with the class. Descriptive statistics were calculated for all items, and exploratory inferential analyses using chi-square tests were conducted to examine differences between grade groups and between participants who attended the MEdit class and those who did not. Free-response comments were analysed using qualitative content analysis by two independent researchers.

**Results:**

Positive responses were reported for understanding of the university (99.6%), interest in medicine (100%), and perceived usefulness for career development (99.6%). Analysis of free-response comments identified four main categories: the significance of practical experience, deepening professional understanding, emergence of inquiry-based thinking, and suggestions for improvement. Exploratory inferential analyses did not identify statistically significant differences in the primary outcome measures between grade groups or between participants who attended the MEdit class and those who did not.

**Conclusions:**

The high school-university collaborative programme implemented by Juntendo University may contribute to enhancing learning motivation and professional understanding among students aspiring to enter medical school. Experiential learning activities and inquiry-based educational approaches may support early career awareness and reflective thinking about the medical profession.

## Introduction

In Japan, the growing demand for healthcare due to super-ageing presents an urgent challenge: cultivating personnel capable of sustaining high-quality medical care. Whilst the number of applicants to medical schools is increasing, challenges remain in fostering an understanding of the roles and qualities required of physicians, and in developing a sense of professional mission. How to provide educational opportunities that enhance career understanding and learning motivation for prospective medical students is a crucial issue in medical education. Curriculum design in medical education is fundamentally based on the learner-centred educational planning proposed by Harden^1)^. Furthermore, Gruppen et al. have highlighted that the learning environment influences the formation of professional identity^2)^. In Japan, university entrance examinations continue to place strong emphasis on academic aptitude scores, and medical school admissions are no exception. However, cultivating students with the qualities needed to become high-calibre physicians and medical researchers should not be confined to post-admission medical school education; systematic engagement from the secondary school and higher secondary education stages is essential. Providing educational opportunities that deepen understanding of the medical profession at an early stage^3)^ and foster purpose and learning motivation is crucial^4)^. Within this context, “high school-university collaboration” initiatives, where secondary schools and universities jointly conduct educational activities, are being promoted across the country. This collaboration aims to enhance students' academic curiosity and career awareness by exposing them to university education, research, and professional activities. It is positioned as a practical framework embodying the principles of the Ministry of Education, Culture, Sports, Science and Technology's “High School-University Connectivity Reform”^5)^. Furthermore, recent years have seen growing demands for enhanced pre-university education and career guidance. OECD reports also emphasise the cultivation of talent in STEM and medical fields as key priorities in education policy^5, 6)^.

Internationally, pipeline programmes and widening participation programmes targeting prospective medical students have been systematically developed, with reported improvements in learning readiness and motivation to pursue higher education^3, 4, 7, 8)^. These initiatives are also considered to reduce mismatches with the educational environment and help prevent student dropout^9)^. In light of these international trends, Juntendo University has designed and implemented a composite high school-university linkage programme that simultaneously fosters understanding of the university, career awareness, and motivation. This programme comprises multiple educational modules: (1) hands- on procedural experience at the simulation centre led by healthcare professionals, (2) lectures by faculty members, and (3) the MEdit class, an original educational module. The details of this programme are described in the Materials and Methods section. This approach aligns with recent reports demonstrating the effectiveness of medical humanities education^10, 11)^. However, within Japan, empirical research on systematic career understanding and motivation education programmes targeting prospective medical students remains limited. In particular, few reports have examined the formation processes of learning motivation and professional awareness during the transition from secondary to higher education. This study aimed to clarify changes in career understanding and learning motivation among prospective medical students through the practice of Juntendo University's integrated secondary-higher education collaboration programme. It conducted questionnaire surveys and free-response analysis of participants to explore the educational effects.

## Materials and Methods

### Study design

This study is a cross-sectional study targeting participants in the university-high school collaboration programme implemented by Juntendo University. A questionnaire survey was conducted immediately after programme participation to quantitatively and qualitatively assess participants' perceptions regarding their understanding of occupations and learning motivation.

### Programme description

The high school-university collaborative programme implemented by Juntendo University consists of several educational modules designed to promote career understanding and learning motivation among prospective medical students. The programme includes: (1) hands-on procedural experience conducted by healthcare professionals at the university simulation centre, (2) lectures delivered by faculty members introducing medical careers and university education, and (3) the MEdit class.

The MEdit class is an original educational module based on the concept of “Medicine × Edit”. It adopts a STEAM-oriented educational approach to explore the relationship between medicine, healthcare, and society from multiple perspectives. The class is designed to encourage inquiry-based thinking and reflection on the nature of diagnosis and medical practice through interdisciplinary discussion and experiential learning activities.

### Participants and study period

Participants included 252 students who took part in the high school-university collaborative program held at Juntendo University Hongo Campus between March 2023 and December 2024. Participants consisted of junior high school and high school students, all of whom participated in part or all of the program activities.

### Data collection

Immediately after completion of the program, participants were provided with a QR code and asked to complete an online questionnaire using Google Forms. Responses were collected anonymously, and each participant was allowed to submit the questionnaire only once to prevent duplicate responses.

The questionnaire covered the following seven areas: (1) History of Open Campus participation ( ‘Was unaware’, ‘Participated online’, ‘Participated on campus’, ‘Could not participate’), (2) Overall enjoyment of the event (4-point scale: ‘Very enjoyable’ to ‘Not at all enjoyable’), (3) Deepening of understanding of the university (4-point scale: ‘Deepened significantly’ to ‘Did not deepen at all’), (4) Change in interest in medicine (4-point scale: ‘Increased significantly’ to ‘Did not increase at all’), (5) Usefulness for career development (4-point scale: ‘Very useful’ to ‘Not useful at all’), (6) Participation in and satisfaction with the MEdit class (Participation: Yes/No, Satisfaction: 5-point scale: ‘Very satisfied’ to ‘Very dissatisfied’), (7) Free-response section (impressions, learning points, improvement suggestions, etc., optional). Each question was rated on a 4-5 point Likert scale, with the free-response section being optional. Participation pathways (e.g., recommendation by school teacher, school noticeboard, recommendation by friend/acquaintance, distributed leaflet, university leaflet, internet search) were also collected via multiple- choice. All questions were multiple-choice format, with only the free-response section requiring text answers. There were no missing responses, and all 252 cases were analysed

### Statistical analysis

Descriptive statistics were calculated for all questionnaire items and are presented as frequencies (n) and percentages (%).

Inferential analyses were conducted to examine differences between grade groups and between participants who attended the MEdit class and those who did not. Grade groups were categorized as junior high school students and high school students.

Associations between categorical variables were analyzed using chi-square tests. When expected cell counts were small, Monte Carlo simulation (B = 20,000) was applied to estimate p-values.

Effect sizes were calculated using Cramér’s V. Statistical significance was defined as p < 0.05.

All statistical analyses were performed using Microsoft^®^ Excel^®^ 2021 (Microsoft Corporation, Redmond, WA, USA).

### Qualitative analysis of free-response comments

Of the 206 free-response comments, 161 were deemed contentually valid and analysed. Following content analysis procedures, the responses were segmented into meaningful units, coded, and similar codes consolidated to extract higher-level categories. Analysis was conducted independently by two researchers, with discrepancies resolved through consultation. There were 206 free-response entries, with 161 included in the analysis. The procedure followed the qualitative evaluation of outreach activities outlined in Brown et al.'s paper^12)^.

### Ethical considerations

This survey was conducted as part of an educational activity and was completely anonymous. No personally identifiable information was collected, and participation was voluntary. The purpose of the study and the policy regarding data use were explained verbally and in written form. Submission of the completed questionnaire was regarded as provision of informed consent.

According to institutional policy and national ethical guidelines, this study was determined not to constitute medical research involving human subjects and was therefore exempt from formal ethical review.

## Results

### Participant characteristics

All 252 participants provided valid responses (response rate: 100%). The participants included 46 junior high school students (18.3%), 106 first-year high school students (42.1%), 66 second-year high school students (26.2%), and 25 third-year high school students (9.9%).

### Enjoyment of the event

**Figure 1:**
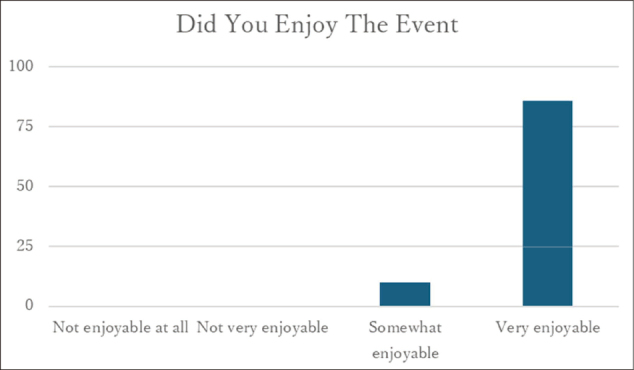
Distribution of responses for the evaluation item “Enjoyment of the event” (n = 96)

Among 96 respondents, all evaluated the event positively, with 89.6% reporting that it was “very enjoyable” and 10.4% reporting that it was “somewhat enjoyable” (Figure 1).

### Understanding of the university

**Figure 2:**
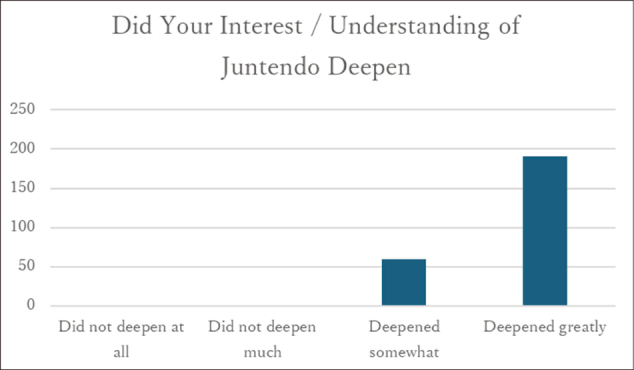
Responses regarding “Deepening understanding of the university” (n = 252)

All 252 participants responded to this item, and 99.6% reported that their understanding of the university had deepened as a result of participation in the program (Figure 2).

### Interest in medicine

**Figure 3:**
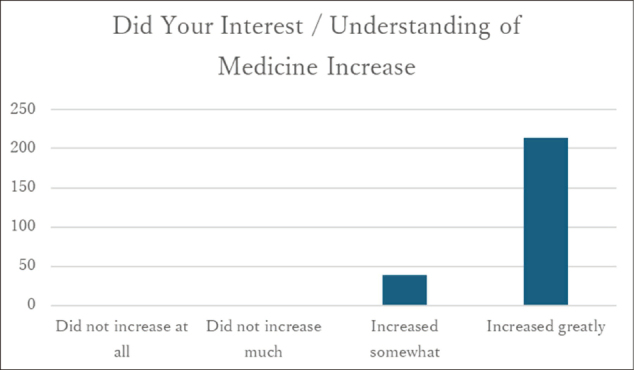
Responses regarding “Changes in interest toward medicine” (n = 252)

All respondents (100%) reported that their interest in medicine had increased after participating in the program (Figure 3).

### Perceived usefulness for career development

**Figure 4:**
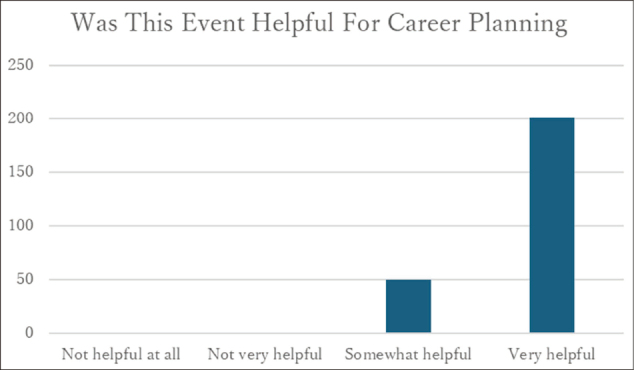
Responses regarding “Usefulness for career development” (n = 252)

Of the 252 participants, 251 (99.6%) reported that the program was useful for their career development (Figure 4).

### Participation in and satisfaction with the MEdit class

**Figure 5:**
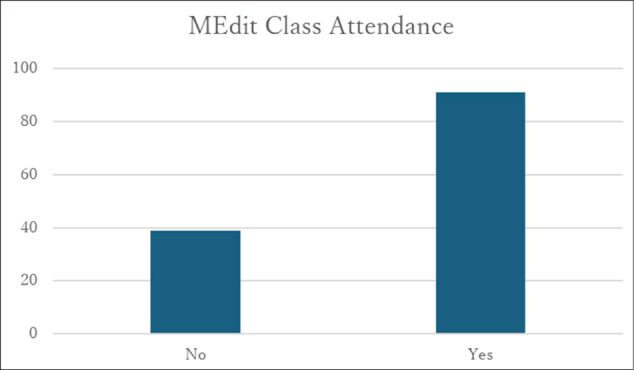
Experience of participating in MEdit classes

**Figure 6:**
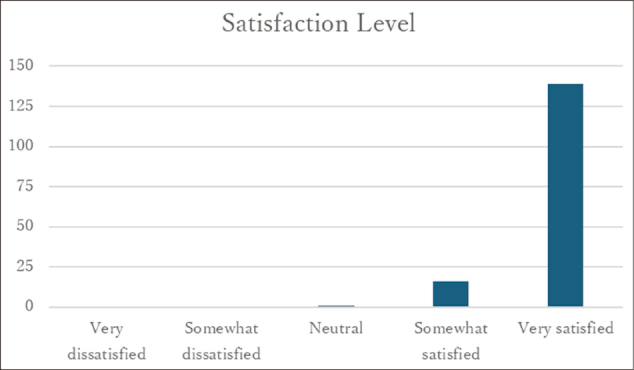
Satisfaction with MEdit classes” (n = 139)

Among 130 respondents who answered questions regarding participation in the MEdit class, 70% reported having participated (Figure 5). Satisfaction with the MEdit class was reported by 139 respondents, of whom 99.3% indicated positive satisfaction (Figure 6). Free-text responses frequently described the class as an opportunity to experience medical thinking and to reflect on the nature of diagnosis.

### Inferential analysis

Exploratory comparisons by grade level were examined; however, due to the extremely high proportion of positive responses across groups, meaningful statistical differences could not be identified.

Comparisons between participants who attended the MEdit class and those who did not showed no statistically significant differences in the primary outcome measures.

### Summary of free-response comments

From the 206 free-response comments, 161 were analysed to extract four categories: 1) the significance of practical experience, 2) deepening understanding of occupations, 3) the emergence of inquiry-based thinking, and 4) other observations and operational aspects.

Significance of practical experience: 53 responses (25.7%) noted that simulation techniques and visits to the medical history museum made learning content tangible. Example: ‘Experiencing the techniques first-hand made me feel the tension and sense of responsibility.’

Deepening professional understanding: descriptions highlighting a realisation of healthcare professionals' roles and the importance of multidisciplinary collaboration (20 cases, 9.7%). Example: ‘I understood that it is the entire team, not just the doctor, who supports the patient.’

Emergence of inquisitive thinking: descriptions indicating that the MEdit class prompted a re-examination of the essence of diagnosis and the relationship between medicine and society (6 cases, 2.9%). Example: ‘By considering diagnostic criteria using a banana as a subject, I contemplated “what diagnosis is”.’

Improvement proposals: operational requests such as extending experience time, adjusting participant numbers and flow, and adding explanatory content (14 cases, 6.8%). Example: ‘I wish the practical session were a bit longer,’ ‘A flow plan tailored to participant numbers is needed.’

Note: the percentages are based on the total number of responses, which allowed multiple categories to be assigned, hence the total does not equal 100%.

## Discussion

This study analyzed 252 participants in the high school-university liaison programmed implemented by Juntendo University. 99.6% of participants reported a deepening understanding of university life, 100% reported increased interest in medicine, and 99.6% responded positively regarding the programmer’s usefulness for career development. The MEdit class achieved a 70% participation rate, with satisfaction levels of “very satisfied” or “somewhat satisfied” accounting for 99.3%. Analysis of open-ended responses identified four categories: “significance of practical experience”, “deepening understanding of professions”, “emergence of inquiry-based thinking”, and “improvement proposals and other comments”. These results suggest that experiential programmed at the secondary school level have the potential to strengthen learners' cognitive foundations regarding career choices.

In addition to the descriptive findings, exploratory inferential analyses were conducted to examine potential differences between grade groups and between participants who attended the MEdit class and those who did not. Although statistically significant differences were not observed in the main outcome measures, this result may reflect the uniformly high level of positive responses across participants. The absence of strong between-group differences suggests that the educational benefits of the programme may be broadly distributed among students regardless of grade level or participation in specific modules.

Previous studies have reported that early experiential placements, particularly early clinical exposure (ECE), contribute to enhancing understanding of healthcare teams, ethical judgement, and motivation to learn^13)^. Such placements serve as an effective bridge between basic medical science and clinical education, contributing to student motivation and the formation of professional perspectives^4)^. While improvements in students' professional awareness and communication skills have been demonstrated, challenges such as insufficient teaching resources and the standardization of assessment methods have been highlighted^14)^. Early exposure programmed for undergraduate students have also been shown to deepen clinical understanding and enhance learning motivation^15)^, revealing a consistent trend that experiential learning influences learners' professional aspirations.

Participants in our university's high school-university linkage programmed gained a deeper understanding of universities and healthcare, increased interest in medicine, and recognized its usefulness for career formation. These results suggest that the educational benefits of early exposure demonstrated in prior research may also be observable at the earlier high school stage.

This program is characterized by incorporating the educational elements of early clinical exposure (ECE) into the high school level. Previous research has primarily examined ECE for medical students, and to our knowledge, studies empirically examining the effects of medical educational ECE for high school students are extremely limited. This research fills this important gap. Such interventions for high school students are also gaining attention within the context of widening participation aimed at reducing educational access disparities^16)^ and are internationally positioned as educational policies that secure career choice opportunities at an early stage. Notably, reports indicate effects on career choices and retention in healthcare professions through long-term follow-up^17)^, highlighting the importance of evaluating the lasting impact of early educational interventions. Participants' high evaluations of the program's usefulness in understanding universities, fostering interest in medicine, and career development suggest that experiential learning can be associated with cognitive and affective learning outcomes even at earlier stages before medical school admission.

Exposing high school students to the realities of healthcare professions and the university educational environment can serve as a catalyst for shifting their career decisions away from traditional, test-score-centric approaches toward more proactive choices grounded in career development, understanding of the profession, and motivation formation. Furthermore, in medical education research, learners making career choices early on based on their own values and motivations is considered a crucial element supporting Professional Identity Formation (PIF). It has been reported that learners with more developed PIF are more likely to exhibit proactive learning attitudes, resilience, and the establishment of ethical judgment^18)^. These factors are thought to contribute to their growth as future healthcare professionals. Furthermore, the holistic review approach adopted in U.S. medical school admissions emphasizes selection based not only on academic ability but also on motivation, values, and experience^19)^, aligning with the importance of motivation-based career formation demonstrated in this study. The improvements in university understanding, career understanding, and motivation to pursue higher education shown in this study may help form this foundational motivational basis at an early stage.

The MEdit class included in this program incorporates elements of medical humanities education and STEAM-based learning, addressing medicine, healthcare, and society in an interdisciplinary manner. Meta-reviews and qualitative studies indicate that medical humanities education contributes to the development of critical thinking, empathy, ethical awareness, and multifaceted perspectives^10, 11)^. Furthermore, arts and humanities-based curricula promote the formation of professional identity (PIF)^20)^. Furthermore, efforts to integrate healthcare simulations into high school education are also implemented overseas as part of STEM/healthcare education^21)^, and have been reported to contribute to stimulating learner interest and enhancing self- efficacy. The practical experience in this program yielded results consistent with the findings and direction of this prior research.

Moreover, many open-ended responses indicated that lecture content exposed students to the essence and values of healthcare, suggesting that the educational approach integrating experiential learning with humanities-based learning may have influenced high school students' cognitive and affective learning processes.

The findings of this study align with previous reports^10)^ demonstrating that medical humanities education contributes to deepening learners' understanding and shaping their values, theoretically supporting the significance of incorporating humanities learning elements into pre-medical education. Furthermore, the program's structure aligns with the principles of “multifaceted thinking” and “formation of professional attitudes” advocated by the Medical Education Model Core Curriculum^22)^, indicating one direction for expanding educational design during the transition from high school to university. Future research should longitudinally track program participants to examine long-term outcomes beyond admission to medical school, including academic performance, learning motivation, development of professional identity formation (PIF), and career choices.

### Limitations

This study has several limitations. First, as a cross-sectional survey, it cannot establish causality regarding whether program participation directly contributed to improved understanding of the university, interest in medicine, or career awareness. Second, all responses are self-reported, making it difficult to completely eliminate social desirability bias or selection bias based on participant characteristics. Third, the content analysis of open-ended responses was exploratory and may not comprehensively capture learners' deep cognitive changes or the developmental process of professional identity. Furthermore, this study did not conduct follow-up surveys after enrollment, leaving the extent of the sustained impact of high school educational interventions on university enrollment, academic achievement, professional identity development, and career choices as a topic for future research.

However, this study employed a cross-sectional design and did not establish causality. Limitations include the lack of post-enrollment follow-up and reliance on self-reported data. Future comparative studies using control groups and longitudinal research are needed to verify the sustainability of educational effects^12, 14)^.

A further limitation of this study is the presence of a potential ceiling effect in several key outcome measures. The proportion of positive responses was extremely high across items, including interest in medicine, understanding of the university, and perceived usefulness for career development. This limited variability may have reduced the discriminative capacity of the measures and the ability to detect meaningful differences between subgroups. Consequently, the absence of statistically significant differences in the comparative analyses should be interpreted with caution, as it may reflect measurement saturation rather than true equivalence between groups.

## Conclusion

This study suggests that the high school-university linkage program implemented by Juntendo University may be associated with improvements in university understanding, interest in medicine, career understanding, and motivation to pursue medical studies among students aspiring to enter medical school. Content from open-ended responses suggested that practical experiences concretized learners' career understanding and sense of responsibility, while inquiry-based learning centered on MEdit classes fostered multifaceted perspectives on healthcare and society. These results indicate that experiential programs at the high school level can serve as an educational foundation supporting the initial stages of future career development as healthcare professionals.

## Data availability

Not applicable. No datasets were generated or analysed during the current study.

## Author contributions

TO led the study, designed the study, and wrote the manuscript. YN provided academic supervision and advice throughout the study. MS performed data analysis and evaluation. KO and HO conducted the high school-university collaborative program with TO and were responsible for data collection. YT supervised the study and provided critical advice on the manuscript.

## Conflicts of interest statement

The authors declare that there are no conflicts of interest.
